# DEAD/H-Box Helicases in Immunity, Inflammation, Cell Differentiation, and Cell Death and Disease

**DOI:** 10.3390/cells11101608

**Published:** 2022-05-11

**Authors:** Parimal Samir, Thirumala-Devi Kanneganti

**Affiliations:** Department of Immunology, St. Jude Children’s Research Hospital, Memphis, TN 38105, USA; fnparima@utmb.edu

**Keywords:** DEAD/H-box proteins, stress granules, chemotherapy, MAP kinase signaling, PRR, PAMP, DAMP, DDX3X, RIG-I, inflammasome, NLR, NLRP3, caspase, interferon signaling, viral infection, bacterial infection, programmed cell death, pyroptosis, apoptosis, necroptosis, PANoptosis, innate immunity

## Abstract

DEAD/H-box proteins are the largest family of RNA helicases in mammalian genomes, and they are present in all kingdoms of life. Since their discovery in the late 1980s, DEAD/H-box family proteins have been a major focus of study. They have been found to play central roles in RNA metabolism, gene expression, signal transduction, programmed cell death, and the immune response to bacterial and viral infections. Aberrant functions of DEAD/H-box proteins have been implicated in a wide range of human diseases that include cancer, neurodegeneration, and inherited genetic disorders. In this review, we provide a historical context and discuss the molecular functions of DEAD/H-box proteins, highlighting the recent discoveries linking their dysregulation to human diseases. We will also discuss the state of knowledge regarding two specific DEAD/H-box proteins that have critical roles in immune responses and programmed cell death, DDX3X and DDX58, also known as RIG-I. Given their importance in homeostasis and disease, an improved understanding of DEAD/H-box protein biology and protein–protein interactions will be critical for informing strategies to counteract the pathogenesis associated with several human diseases.

## 1. Introduction

**Figure 1:**
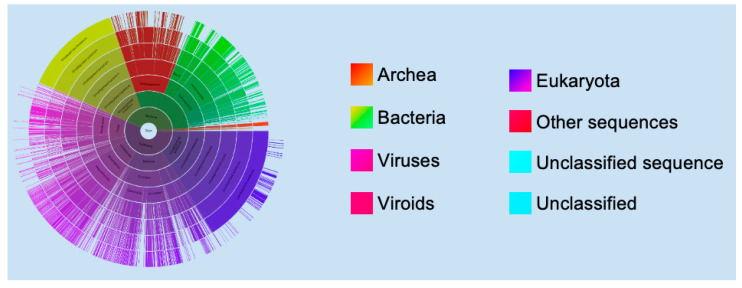
**Distribution of DEAD/H-box domain-containing proteins.** The sunburst plot was downloaded from the Pfam database [8]. In total, 8874 species are represented in the sunburst plot. The Pfam database has 171,615 protein sequences and 2493 domain architectures that contain the DEAD/H-box domain. Arc lengths in the sunburst plot are dependent on the number of protein sequences in each category. A vast majority of DEAD/H-box-containing protein sequences are from eukaryotes (purple), and none are found in viruses. The original interactive sunburst plot can be viewed at the Pfam database using the following link: https://pfam.xfam.org/family/PF00270#tabview=tab7 (accessed on 20 July 2021).

RNA helicases play a central role in RNA metabolism, affecting many aspects of cellular homeostasis. DEAD/DEAH (DEAD/H)-box helicases make up the largest family of RNA helicases in humans [1]. The DEAD/H-box helicases are present in every kingdom of life (Figure 1), and their essential role in translation was discovered in the late 1980s [2,3,4,5,6]. Since then, they have been found to be involved in almost every aspect of cell biology, including splicing, RNA metabolism, host responses to bacterial and viral infections, organismal development, stress responses, and programmed cell death (PCD).

DEAD/H-box helicases have a domain architecture containing the conserved amino acid motifs Asp-Glu-Ala-Asp (DEAD) or Asp-Glu-Ala-His (DEAH), and the Helicase_C sequence motif. There are 199 human and 116 mouse protein sequences that contain the DEAD/H-box domain in the InterPro database [7]. Additionally, there are 1913 domain architectures involving the DEAD/H-box domain (PFAM accession number: PF00270), the vast majority of which contain both a Helicase_C domain (PFAM accession number: PF00271) and low-complexity or disordered regions [8]. Furthermore, there are 3268 domain architectures containing the Helicase_C domain, with the most common architectures also containing the DEAD/H-box domain. Phylogenetic analysis shows that most of the DEAD/H-box proteins have orthologs in both humans and mice (Supplementary Data S1). This conservation suggests that the information gleaned from studies of these proteins in mouse models may be extrapolated to their roles in humans. Additionally, some of the DEAD/H-box proteins have important paralogs whose relative contributions in biological processes remain poorly understood. An example of one such paralogous relationship can be found between DDX3X, DDX3Y, and DDX3L (also known as D1PAS1) in mice (Supplementary Data S1). DDX3X is one of the most extensively studied DEAD/H-box proteins, though there is still much to learn about DDX3Y and DDX3L. These relationships will be discussed in more detail later in this review. Understanding the functions of the paralogs will be critical for precise therapeutic targeting to avoid unintended adverse events.

In mammals, several DEAD/H-box proteins act as prototypical nucleic acid-sensing pattern recognition receptors (PRRs), as proposed by Janeway [9]. DEAD/H-box proteins have also been associated with a range of human diseases (Supplementary Data S2). These observations bolster the argument for in-depth studies of the DEAD/H-box family of proteins. In this review, we discuss the state of knowledge regarding DEAD/H-box proteins, with a focus on their known roles in the host response to pathogenic challenges, PCD, and disease.

## 2. DEAD/H-Box Proteins and the Innate Immune Response to Viral Infections

**Figure 2:**
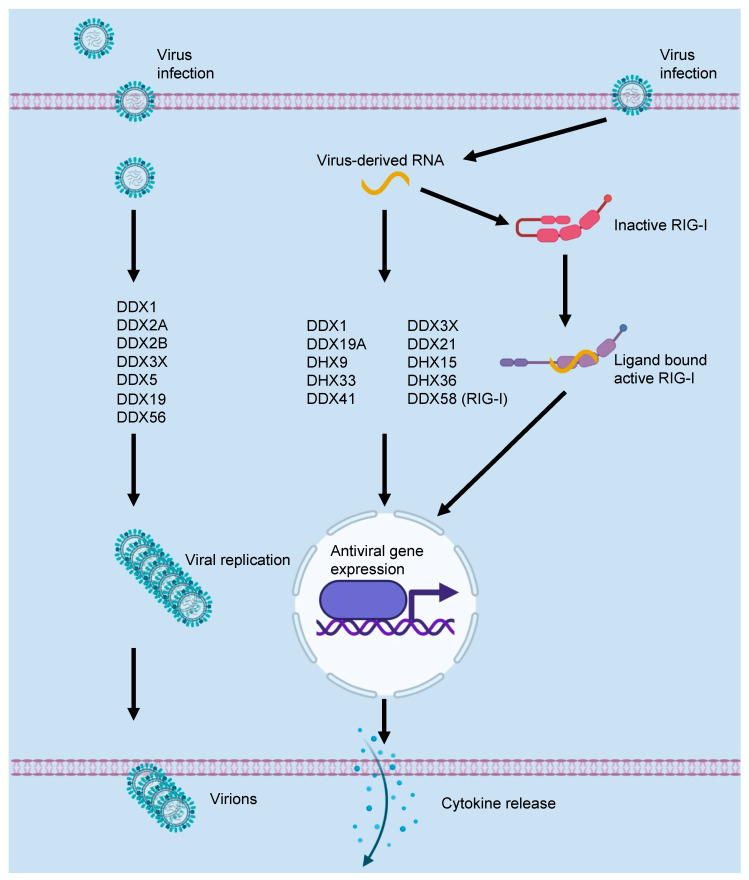
**Roles of DEAD/H-box proteins in viral replication and antiviral host responses.** DEAD/H-box proteins play pleiotropic roles in host responses to viral infections. Some DEAD/H-box proteins promote viral replication through their functions in transcriptional, post-transcriptional, or translational control of gene expression. Some DEAD/H-box proteins act as viral nucleic acid sensors and promote an antiviral host response. Additionally, some have both capabilities. RIG-I (also known as DDX58) is a prototypical example of a DEAD/H-box protein acting as a nucleic acid sensing pattern recognition receptor.

DEAD/H-box proteins play an important role in many host RNA metabolic processes, and they are often referred to as ribonucleoprotein particle (RNP) modulators [10]. Due to their diverse functions in RNA metabolism, they aid in the host response against viral infection (Figure 2). However, viruses have evolved the ability to use DEAD/H-box proteins for replication. One key example of this ability can be seen in the case of the first DEAD/H-box protein discovered—eukaryotic initiation factor 4A (eIF4A), also known as DDX2 [6]. In one of the earliest reports of a DEAD/H-box protein having a role in the viral life cycle, eIF4A was found to bind Semliki Forest virus mRNA [11]. Subsequent studies have shown that eIF4A binds to several viral mRNAs [12,13,14,15], and these studies have made critical contributions to our understanding of eukaryotic translation initiation. eIF4A helps to unwind secondary structures in 5′-untranslated regions (5’-UTR) to facilitate ribosome scanning [16,17]. eIF4A is also important for internal ribosome entry site (IRES)-mediated, cap-independent translation initiation [16,17]. Since several viruses have evolved to repress host cellular cap-dependent translation and use IRES-mediated translation for viral protein synthesis, eIF4A has emerged as an attractive target for developing antiviral drugs [15,18,19,20,21,22,23].

Another well-studied example of viral hijacking of host machinery is the X-linked DEAD/H-box helicase, DDX3X. DDX3X was originally identified for its role in the translation of host and viral RNA [24,25,26]. It was initially found to interact with hepatitis C virus (HCV) proteins, but the functional significance of the interaction was unknown at the time [27,28,29]. Follow-up studies showed that DDX3X promotes HCV replication, and DDX3X has since been implicated in promoting replication for influenza A virus (IAV), West Nile virus (WNV), Japanese encephalitis virus, and human immunodeficiency virus (HIV) [30,31,32,33,34,35]. DDX3X is required for the nuclear export of HIV, and it also acts as a negative regulator of type I interferons in arenavirus infections [30,31,36]. Though these studies suggest that DDX3X inhibition could be a potential antiviral therapeutic strategy, DDX3X has dual functionality in that it can promote both viral replication and optimal antiviral host response [34,35,37,38,39,40,41]. For example, DDX3X facilitates HIV replication [31], but it also acts as a sensor for aborted HIV transcriptomes [42]. Its importance for both the host and pathogen has made this conserved protein one of the most well-studied DEAD/H-box proteins.

Both the murine *Ddx3x* and human *DDX3X* genes are capable of complementing the deficiency of the *Ded1* yeast ortholog [25,27]. This conservation may help to explain DDX3X’s dual role in host–pathogen interactions. Viruses have coevolved with organisms expressing a DDX3X ortholog for millions of years. This may have allowed them to develop strategies to blunt DDX3X’s antiviral function and to co-opt its role in gene expression and subcellular transport for viral replication. This is reflected in the observations that several viruses can target DDX3X to inhibit the induction of the type I interferon response [35,41,42,43,44,45,46]. As viruses have evolved to inactivate the DDX3X-mediated type I interferon response, hosts have similarly found ways to engage other sensors and signaling pathways, or to acquire new functions for existing sensors. For example, DDX3X can promote an NLRP3 inflammasome-mediated pro-inflammatory response to IAV infection [47]. DDX3X can also promote inflammation by enhancing the phosphorylation of the protein phosphatase PP2A to activate NF-kB signaling downstream of TLR3 activation [48].

In addition to DDX3X, there are other DEAD/H-box proteins that mediate host–virus interactions. DDX58 (also known as RIG-I) was implicated in the antiviral host response when it was discovered that RIG-I is induced by porcine reproductive and respiratory syndrome virus (PRRSV) infection [49]. While RIG-I was originally proposed to be a double-stranded RNA (dsRNA) sensor [50,51,52], later studies showed that RIG-I recognizes uncapped RNA containing a 5′-triphosphate moiety [53,54]. Since endogenous mRNA, with the exception of mitochondrial and chloroplast mRNA, contains a 5′-7-methylguanosine cap structure, the presence of the 5′-triphosphate moiety on cytoplasmic RNA acts as a pathogen-associated molecular pattern (PAMP) for recognition by innate immune sensors [9,55]. RIG-I is the only DEAD/H-box protein that also contains a caspase activation and recruitment domain (CARD). Activation of RIG-I leads to the recruitment of MAVS (mitochondrial antiviral signaling, also known as IPS-1, interferon-β promoter stimulator 1) through CARD–CARD interactions, which in turn leads to the induction of a type I interferon response [56,57]. RIG-I undergoes a conformational change upon ligand binding [58,59] which makes the CARD domain accessible for interaction with MAVS, and which allows for the TRIM25-mediated generation of free K63-linked polyubiquitin chains. These chains associate with RIG-I CARD domains and help to bring MAVS into a complex with RIG-I. RIG-I has two CARD domains, which means that it has a valency of two for CARD–CARD interactions [60]. Multivalency has recently been proposed to be critical for the assembly of biomolecular complexes involved in innate immune signaling and PCD [60,61,62]. Functionally, the RIG-I bivalency with respect to CARDs could help to bring together different molecules into a single complex, though this remains to be thoroughly investigated. 

Though not as well characterized, many other DEAD/H-box proteins have roles in host–virus interactions. Some have a positive impact on viral replication. For instance, DDX1 is a cofactor for the HIV-Rev protein that promotes viral replication [31]. DDX1 also promotes the replication of avian infectious bronchitis virus (IBV), a model coronavirus, by interacting with non-structural protein 14 (NS14) [63]. The role of DDX1 in the replication of human coronaviruses remains to be determined. If this function of DDX1 is conserved for human coronaviruses, the targeting of DDX1 could be a viable strategy for treating coronavirus infections [64]. DDX1 also forms a complex with two other DEAD/H-box proteins, DDX21 and DHX36, to promote an antiviral type I interferon response through TRIF in myeloid dendritic cells (mDCs) [65]. In this complex, DDX1 binds dsRNA while DDX21 and DHX36 interact with TRIF [65]. The PRK^DDX21^ domain of DDX21 interacts with the TIR^TRIF^ domain of TRIF through heterotypic domain interactions [65]. The interaction between DHX36 and TRIF is also mediated by heterotypic domain interactions between HA1-DUF^DHX36^ and TIR^TRIF^ domains [65]. Whether the DDX1-DDX21-DHX36 complex can form in cells other than mDCs remains to be seen. 

There are several other examples of DEAD/H-box proteins that are involved in the host–pathogen interaction. DDX5 promotes the viral replication of Japanese encephalitis virus by binding to the viral 3′-UTR [66]. DDX19 can associate with both spliced and unspliced IAV mRNAs, and it binds to IAV polymerase independent of RNA and promotes the nuclear export of viral mRNA [67]. Additionally, DDX56 increases the infectivity of WNV through a mechanism that is independent of viral replication [68]. The helicase activity of DDX56 is required for increased infectivity [69]. It was proposed that DDX56 is required for WNV virion assembly. However, the endogenous function of DDX56 remains poorly understood. A recent study showed that DDX56 is a negative regulator of type I interferon signaling, suggesting that it could have a role in suppressing excess inflammation to maintain organismal homeostasis [70]. 

DHX9 promotes antiviral signaling by sensing dsRNA and activating the NLRP9b inflammasome in response to rotavirus infection in intestinal epithelial cells [71], although whether DHX9 is sensing a specific secondary structure in the dsRNA that is acting as a PAMP remains to be seen. In plasmacytoid dendritic cells, DHX9 responds to CpG-B to activate NF-κB and cytokine production, including TNF and IL-6 [72]. Additionally, DDX19A and DHX33 promote activation of the NLRP3 inflammasome in response to viral infections [73,74]. DHX15 is another DEAD/H-box protein that is involved in antiviral innate immune responses. It promotes MAVS- and NLRP6-dependent antiviral signaling in enteric encephalomyocarditis virus (EMCV) infection by facilitating NLRP6 binding to viral RNA [75]. Additionally, DHX15 was found to undergo liquid–liquid phase separation with NLRP6 in response to dsRNA [76]. During EMCV infection, DHX15 also promotes interactions with MAVS to induce interferon production and signaling [75]. Furthermore, DHX15 also binds to RIG-I through a heterotypic interaction with CARD^RIG-I^ and the viral RNA PAMP to promote type I interferon signaling [77]. Induction of the type I interferon response is dependent on RIG-I, and DHX15 binding promotes ATP hydrolysis upon RNA PAMP binding. Since both DHX15 and RIG-I have ATPase activity, it is not clear which protein is responsible for increased ATP hydrolysis. Additionally, DDX41 plays a role in the host response to viral infections. DDX41 can activate STING-dependent type I interferon and NF-κB and MAP kinase signaling upon dsDNA binding in mDCs [78]. DDX41 binds dsDNA and STING simultaneously to activate this downstream signaling [78].

Together, the data suggest that DEAD/H-box proteins, particularly those involved in positively regulating the viral life cycle, can be targeted to inhibit viral replication. However, there is a need for a comprehensive study on functions of DEAD/H-box proteins during viral infections to clearly identify those which positively regulate viral infection. A deeper understanding of this field will expand the repertoire of host factors that can be therapeutically targeted to treat specific viral infections.

## 3. DEAD/H-Box Proteins and the Innate Immune Response to Bacterial Infections

**Figure 3:**
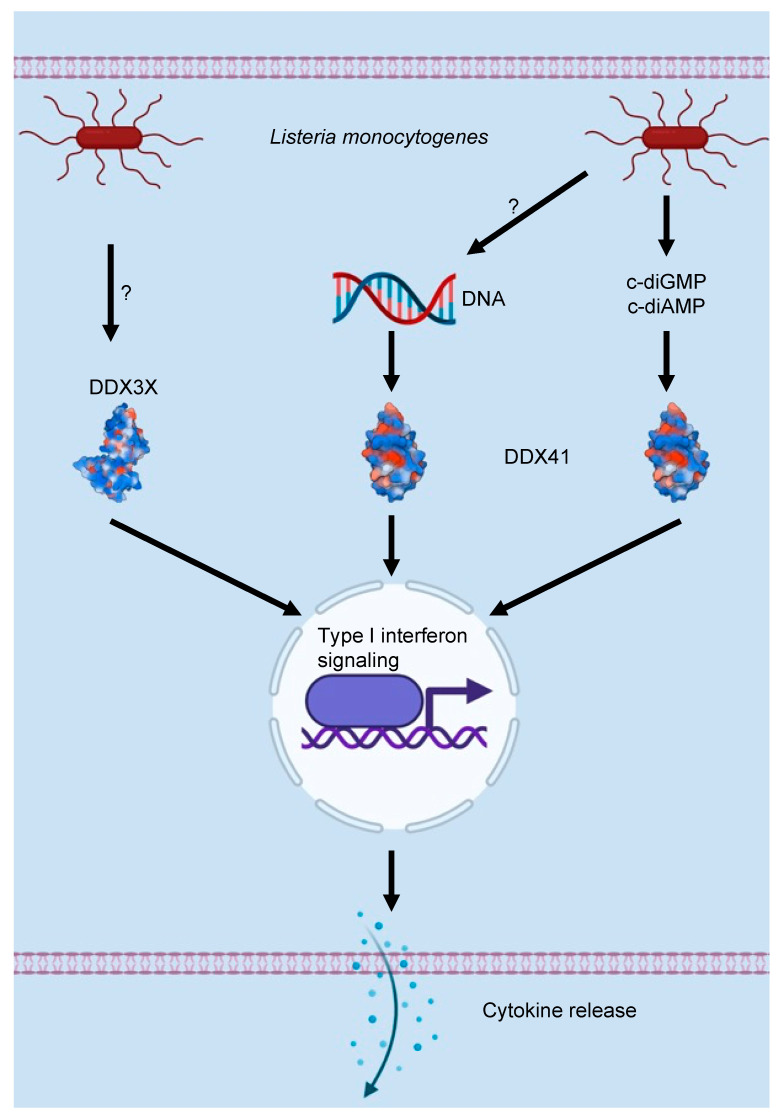
**Role of DEAD/H-box proteins in the host response to bacterial infection.** DDX3X can activate type I interferon signaling in response to infection with *Listeria monocytogenes* downstream of TBK1. Bacterial secondary messengers can also be sensed by hosts to activate the type I interferon response. DDX41 acts as a sensor for c-diGMP and c-diAMP to activate the STING-dependent type I interferon response. DDX41 can also sense DNA, and may be able to sense bacterial DNA during infection. Solvent-excluded surfaces of DDX3X (PDB ID: 2I4I) [83] and DDX41 (PDB ID: 2P6N) [84] are depicted in the figure. Hydrophobic residues (red) and hydrophilic residues (blue) on the surface of proteins are shown.

Although understudied compared to their roles in viral infections, DEAD/H-box proteins also have critical functions in responding to bacterial infections. In response to *Listeria monocytogenes* infection, DDX3X acts as a transcription factor and upregulates the expression of the *Ifnb* gene in macrophages (Figure 3) [79]. DDX3X is phosphorylated by TBK1, which leads to its nuclear translocation and recruitment to the *Ifnb* promoter to activate its transcription [79]. In addition, bacterial secondary messengers can act as PAMPs for recognition by innate immune system PRRs such as DDX41. Cyclic di-GMP (c-diGMP) and cyclic di-AMP (c-diAMP) are ubiquitously present bacterial second messengers that are sensed by DDX41 [80]. DDX41 binds to STING and activates type I interferon signaling upon *L. monocytogenes* infection in the mouse splenic dendritic cell line D2SC (Figure 3). DDX41 can sense DNA to activate signaling [78,80], and cytoplasmic delivery of c-diGMP and c-diAMP also activates the type I interferon response in a DDX41-dependent manner [80]. Bacterial DNA is likely also released from lysed cells, and it can activate the host response independently of DDX41, providing another example of the multifaceted nature of PAMP sensing mechanisms used by mammalian hosts to counteract pathogenic infections [47,80,81,82].

Although DEAD/H-box proteins are canonically classified as RNA-binding proteins, they can bind DNA and have been reported to sense cytosolic DNA [72,78]. While most studies have focused on DEAD/H-box proteins sensing DNA viruses, DNA-sensing DEAD/H-box proteins can also biochemically detect bacterial DNA that is released in the cytoplasm of infected cells. It will be interesting to evaluate whether these DEAD/H-box proteins also have a role in the host response to bacterial infection. Studies to investigate these processes could have important translational impacts and identify new targets for therapeutic interventions in diseases caused by bacterial infections.

## 4. DEAD/H-Box Proteins in Programmed Cell Death Regulation

**Figure 4:**
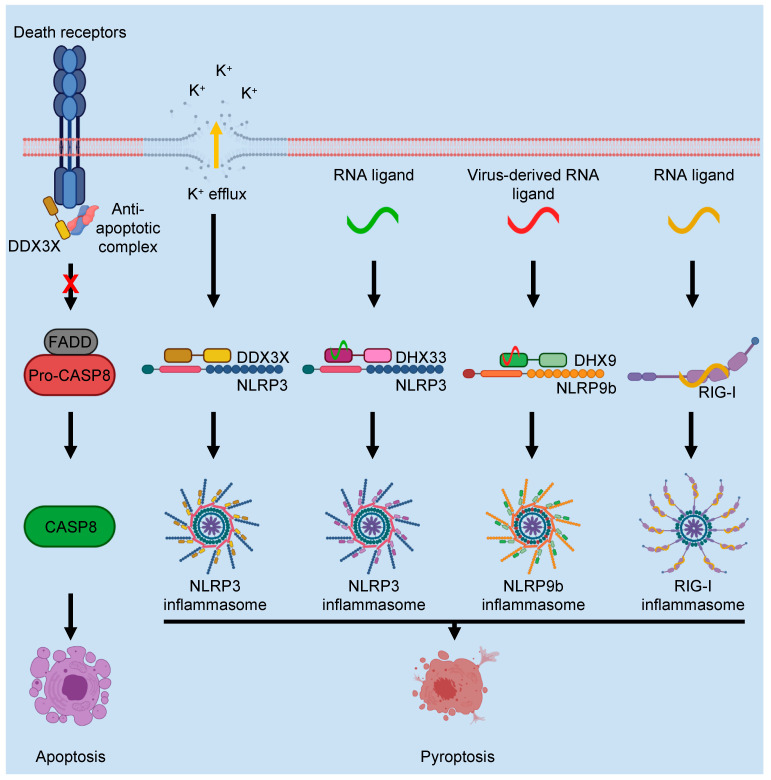
**Role of DEAD/H-box proteins in programmed cell death.** DDX3X forms an anti-apoptotic complex with cIAP and GSK3 that blocks extrinsic apoptosis induced by ligands, such as TNF, TRAIL, and FASL binding to death receptors. Signals that induce apoptosis target DDX3X for caspase-8 (CASP8)-mediated degradation. Several DEAD/H-box proteins have been reported to drive pyroptosis—a pro-inflammatory programmed cell death pathway. DDX3X is critically important for the activation of the canonical NLRP3 inflammasome downstream of potassium ion efflux, as well as potassium-independent mechanisms. Another DEAD/H-box protein that is involved in triggering NLRP3 inflammasome activation is DHX33. DHX33 acts as a sensor of virus-derived dsRNA, and it activates the NLRP3 inflammasome. DHX9 is a sensor for rotavirus-derived dsRNA for activating the NLRP9b inflammasome. RIG-I (DDX58) itself can also act as an inflammasome sensor. Virus-derived RNA or 5′-triphosphate-containing RNA can trigger assembly of an inflammasome dependent on RIG-I, ASC, and CARD9.

The role for programmed cell death (PCD) in organismal development and host responses to pathogenic challenges has been an active area of research for many decades [85,86]. These studies have discovered interlinked PCD pathways that are critical for maintaining organismal homeostasis [81,87,88,89,90,91,92,93,94,95,96,97,98,99,100,101,102,103,104,105,106,107,108,109]. Several DEAD/H-box proteins have been implicated in the regulation of PCD (Figure 4). DEAD/H-box proteins can both promote and inhibit cell death. DDX3X provides a prime example of the contrasting dual roles of DEAD/H-box proteins in PCD (Figure 4). DDX3X can inhibit apoptosis and promote pyroptosis [110,111]. In response to stimulation of the death receptors TRAILR1, TRAILR2, TNFR, and FAS, DDX3X forms an anti-apoptotic complex with GSK3 and CIAP1, which acts as a cap around the cytosolic face of the death receptors and sterically inhibits their activity [111]. This, in turn, inhibits extrinsic apoptosis. Conversely, the inhibition of DDX3X ATPase activity by the small molecule inhibitor RK-33 promotes apoptosis [112]. Additionally, signals that activate apoptosis promote the proteolytic degradation of DDX3X and CIAP1, and DDX3X is a target of CASP8-mediated proteolytic processing [111]. This suggests that a major function of this anti-apoptotic complex guards against the accidental activation of extrinsic apoptosis. However, the molecular events that trigger CASP8 activation to lead to DDX3X cleavage have not been completely elucidated. 

In contrast, DDX3X is required for pyroptosis downstream of NLRP3 inflammasome activation [110]. DDX3X was found to be required for NLRP3 inflammasome activation by both potassium efflux-dependent and -independent triggers, but the exact molecular mechanism is not completely understood. It was proposed that the scaffold function of DDX3X promotes prionoid phase transitions, which lead to the formation of complex assemblies that can act as a platform to trigger cell death. Examples of such assemblies include ASC specks, amyloid plaques, Tau fibrils, and PANoptosomes, which are multifaceted macromolecular complexes that regulate PANoptosis, an inflammatory PCD that integrates components from other cell death pathways; the totality of the biological effects in PANoptosis cannot be individually accounted for by pyroptosis, apoptosis, or necroptosis alone [81,92,93,94,95,96,97,98,99,100,101,102,103,104,105,106,107,108,109]. Since the liquid–liquid phase separation of DDX3X promotes the assembly of stress granules, which are thought to inhibit PCD, it was proposed that, depending on the type of phase transition triggered, DDX3X can promote either cell survival or PCD [110,113]. However, the molecular mechanism by which DDX3X is recruited, either to stress granules or to the NLRP3 inflammasome, requires further study. Taken together, DDX3X has been reported to play a pleiotropic role in PCD, immunologically inhibiting silent apoptosis while promoting inflammatory PCD.

RIG-I is another DEAD/H-box protein that has been extensively studied for its role in PCD (Figure 4). RIG-I induces mitochondrial apoptosis in melanoma and hepatoma cells in response to 5′-triphosphate–containing RNA, as well as the dsRNA analog polyinosinic-polycytidylic acid (poly(I:C)) [114,115]. As discussed above, RIG-I is activated by viral transcripts, and this leads to the induction of the type I interferon response. While PCD induction by RIG-I was initially thought to be independent of type I interferon signaling, more recent work shows that RIG-I can promote apoptosis downstream of IFNα in melanoma cells [116]. Additionally, combinatorial treatment with a STAT3 inhibitor and IFNα increases apoptosis in melanoma cells [116], suggesting that the activation of RIG-I in conjunction with cancer therapy could potentially improve its therapeutic efficacy. RIG-I activation and the induction of apoptosis could also have a beneficial role in photochemotherapy for treating psoriasis [117]. Furthermore, RIG-I has been proposed to induce IRF3-dependent apoptotic cell death in response to viral infections [118,119]. IRF3-dependent apoptosis in Sendai virus-infected cells is dependent on IRF3 binding to the pro-apoptotic protein BAX, and it is activated by signaling downstream of RIG-I-like receptors [120,121]. In addition to its roles in apoptosis, RIG-I was also reported to assemble an inflammasome in bone marrow-derived dendritic cells [122]. This inflammasome activation requires CARD9 and the inflammasome adaptor ASC. Additionally, in human primary lung epithelial cells, RIG-I can assemble an inflammasome in response to IAV infection in a type I interferon signaling-dependent process [123]. These studies suggest that RIG-I may induce pyroptosis in addition to apoptosis. The CARD domain of RIG-I could be responsible for a RIG-I-containing PCD complex upon ligand binding. Although these studies have shed light on RIG-I-mediated apoptosis and pyroptosis, a complete mechanistic understanding of the process remains elusive.

DDX3X and RIG-I are the most well-studied DEAD/H-box proteins with respect to their role in PCD. However, several other DEAD/H-box proteins have also been implicated in PCD (Figure 4). A screen designed to identify DEAD/H-box proteins involved in the activation of the NLRP3 inflammasome found that DHX33 can form a complex with NLRP3 and ASC that promotes caspase-1 (CASP1) cleavage and inflammasome-dependent cytokine processing [73]. DHX33 essentially acts as an RNA sensor to trigger NLRP3 inflammasome activation. Cytosolic poly(I:C), viral dsRNA, and bacterial RNA promote DHX33-mediated activation of the NLRP3 inflammasome [73]. The interaction between NLRP3 and DHX33 is driven by heterotypic domain interactions between DEAD^DHX33^ and NACHT^NLRP3^. This interaction might be mediated by intrinsically disordered regions (IDRs) in DEAD^DHX33^ and NACHT^NLRP3^, in a manner similar to the interaction between DDX3X and NLRP3 [110], but this remains to be tested. DDX19A is another DEAD/H-box protein that has been implicated in NLRP3 inflammasome activation in response to viral infection [74]. PRRSV infection of primary porcine alveolar macrophages activates the NLRP3 inflammasome, and DDX19A is thought to act as an RNA sensor for PRRSV infection [74]. Additionally, DHX9 is a DEAD/H-box protein that is reported to have contrasting roles in PCD. DHX9 acts as a dsRNA sensor in rotavirus-infected intestinal epithelial cells, to facilitate pyroptosis through NLRP9b inflammasome activation [71]. Since DHX9 and NLRP9b do not share a domain for homotypic interactions, their mechanism of interaction is unknown. This may occur through IDRs or with the dsRNA ligand acting as a bridge. In contrast to its role in activating pyroptosis, DHX9 inhibits the lytic replication cycle during infection with Epstein-Barr virus (EBV) [124]. This lytic replication involves PCD, suggesting that DHX9 is a survival factor in EBV-infected cells [125]. 

Overall, DEAD/H-box proteins play diverse roles in regulating PCD in response to pathogenic infections. However, most of the proteins in the DEAD/H-box family are understudied in the context of PCD, suggesting that there are still discoveries to be made. For example, DDX5 has been most intensely studied for its role in splicing, gene expression, and oncogenesis; however, beyond these roles, the phosphorylation of DDX5 can inhibit TRAIL-induced apoptosis in glioblastoma cells [126]. The double tyrosine phosphorylation of DDX5 downstream of platelet-derived growth factor signaling is associated with the inhibition of CASP8 cleavage and resistance to TRAIL-induced apoptosis in T98G glioblastoma cells [126]. This suggests that further studies to evaluate additional DEAD/H-box proteins in PCD may identify other such functions.

## 5. DEAD/H-Box Proteins in Cell Differentiation and Organismal Development

Beyond their roles in infection, PCD and host defense, DEAD/H-box proteins are also critically important for cell differentiation and organismal development. One key example is *Dhx9*, which is one of the earliest DEAD/H-box genes to be discovered. It was originally identified through the observation of a temperature-sensitive mutation in *Drosophila melanogaster* that led to defective nerve conduction at the restrictive temperature, suggesting a role in neuronal development [127]. Researchers named the gene *no action potential* (*Nap*). Two other groups independently identified mutations in the same gene that caused male sterility in *D. melanogaster* and referred to the gene as *maleless* (*Mle*) [128,129]. The human ortholog *DHX9* was identified from HeLa cells and was initially named *RNA Helicase A* [130]; at the time, it was not classified as a DEAD/H-box protein because the characteristic DEAD/H-box sequence motifs had not yet been discovered. Soon after, *RNA Helicase A* and *Mle* were found to be orthologs, and they were classified as members of the DEAH family of DEAD/H-box proteins, leading to their subsequent renaming as *DHX9* [131]. In *D. melanogaster*, *Dhx9* was found to be important for the proper activation of X-chromosome dosage compensation; its dysfunction led to male sterility. In contrast to *D. melanogaster Dhx9*, murine *Dhx9* is required for normal gastrulation in both male and female embryos [132]. Additionally, there is increased apoptosis in *Dhx9*^−/−^ mouse embryos compared with the wild type [132], further supporting a role for DHX9 in PCD in addition to organismal development. 

DDX17 is another DEAD/H-box protein that has been reported to play a role in cell differentiation and organismal homeostasis. *Ddx17* was also discovered in *D. melanogaster* and was originally named *Lighten up* (*Lip*) [133]. DDX17, along with the DEAD/H-box protein DDX5, is involved in regulating alternative splicing during epithelial cell and myotube differentiation in mammals [134]. The sensing of endogenous short interspersed nuclear elements by DDX17 is associated with the assembly of an inflammasome containing NLRP3 and NLRC4 that does not induce pyroptosis but does drive cytokine release, contributing to disease in a murine model of atrophic macular degeneration and IL-18 release in cells from patients with systemic lupus erythematosus [135]. 

DDX3X and its paralog, DDX3Y, have also been reported to regulate organismal development [136]. In mice, the loss of *Ddx3x* during hematopoiesis results in an abnormal leukocyte composition in the bone marrow and spleen [137]. Additionally, RIG-I is essential for myelopoiesis through its regulation of the TRIM25-dependent protein ISGylation [138]. Other DEAD/H-box helicases have also been implicated in hematopoiesis in other model systems, including DDX46 and DDX18 in zebrafish [139,140]. Beyond hematopoiesis, the loss of X-linked *Ddx3x* in mice also leads to female-specific defects in hindbrain development [136]. Males are presumably protected because of the presence of Y-linked *Ddx3y*. DDX3X is involved in neuronal development in humans as well [141,142,143]. Mutations in *DDX3X* lead to RNA metabolism defects that are associated with intellectual disability (discussed in more detail in the next section) [143]. These studies suggest that DEAD/H-box proteins can play critical roles in cell differentiation and organismal development. However, they have been comparatively understudied in this context, due to a lack of in vivo models, likely due to the high chance of embryonic lethality upon complete deletion. In recent years, conditional deletions in specific subsets of cells have allowed scientists to begin to evaluate the role of more DEAD/H-box proteins during development [136]. Recent developments in genome editing technologies, including conditional deletion and activation technologies, should be beneficial for future studies of DEAD/H-box proteins in the context of cell differentiation and organismal development.

## 6. DEAD/H-Box Proteins in Human Diseases

Owing to their diverse role in RNA metabolism and organismal homeostasis, it is not surprising that aberration in the functions of DEAD/H-box proteins have been implicated in a range of human diseases. For instance, the dysregulation of DEAD/H-box helicases is linked to hematological malignancy, as well as to cancer growth and metastasis. One relatively well-characterized connection between DEAD/H-box proteins and disease is Bloom syndrome, an autosomal-recessive disorder that leads to stunted growth and genomic instability [144,145,146]. The gene associated with Bloom syndrome was mapped to human chromosome 15 and later identified as a DEAD/H-box helicase, *BLM* *RecQ-like helicase* (BLM) [146,147]. BLM inhibits double-strand breaks during DNA replication that can increase cancer predisposition in cell lines [148]. Consistent with these in vitro findings, *Blm*^−/−^ mice have an increased predisposition to develop lymphoma, sarcoma, and carcinoma [149]. Since the discovery of the link between BLM and Bloom syndrome, several human gene–disease associations (GDAs) have been discovered involving DEAD/H-box proteins. For example, loss-of-function mutations in *Werner syndrome ATP-dependent helicase* (*WRN*) lead to Werner syndrome, a disease that is characterized by premature aging and increased susceptibility to cancer. *WRN* was discovered through positional cloning and was later found to have DNA helicase activity [150,151]. Subsequent studies showed that WRN is involved in resolving Holiday junctions during DNA recombination, the unwinding of DNA secondary structures, and DNA repair [152,153,154,155]. A recent study suggested that WRN is also involved in the nuclear export of mRNA [156].

In addition to *BLM* and *WRN*, mutations in *DDX3X* have been associated with several diseases, specifically several cancers, epilepsy, and female intellectual disability [157,158,159,160,161]. Multiple specific mutations in *DDX3X* are associated with cancer (Supplementary Data S2). However, there is conflicting evidence regarding the role of DDX3X in tumorigenesis and patient survival. Low levels of *DDX3X* expression have been associated with poor prognosis in patients with colorectal cancer [160] and in patients who are non-smokers with oral cancer [162], while a high expression of *DDX3X* is associated with poor prognosis in gliomas [163]. Other studies have reported an oncogenic role for DDX3X in colorectal cancer [159,164]. Additionally, *DDX3X* depletion has been reported to reduce metastasis in medulloblastoma [165], while this depletion leads to increased malignancy in non-small cell lung cancer [166]. These studies suggest an urgent need to clarify the role for DDX3X in cancers, as it has been proposed as a target for developing anti-cancer therapeutics [112,167,168,169]. Mechanistically, cancer-associated *DDX3X* mutants have reduced RNA-dependent ATPase activity, and their expression leads to increased stress granule assembly [170,171]. These findings support the hypothesis that reduced translation can promote tumorigenesis [171]. Since translation is the most energy-intensive process in a cell, a reduced rate of translation caused by *DDX3X* mutations might allow for the metabolic adaptation of cancer cells for their continued proliferation [172,173]. Additionally, mutations in the *DDX3X* paralog *DDX3Y* are also associated with tumors of the reproductive system [174,175]. Loss-of-function mutations in *DDX3Y* result in male sterility, again suggesting a complex role for DDX3X/Y in cancer [176]. 

Overall, DEAD/H-box protein GDAs span human diseases and include intellectual disability, cancers, susceptibility to viral infections, and behavioral defects, among others. An analysis of DEAD/H-box protein GDAs from the DisGeNET database highlights the depth and breadth of these associations (Supplementary Data S2) [177]. Overall, the diversity of DEAD/H-box protein GDAs is indicative of the critical role that DEAD/H-box proteins play in maintaining organismal homeostasis. This importance draws attention to the need for a concerted effort to mechanistically characterize DEAD/H-box protein functions to understand their specific roles in each disease, and to identify new therapeutic strategies. 

## 7. Discussion

DEAD/H-box proteins constitute the largest family of RNA helicases in mice and humans. Beyond the many roles for DEAD/H-box proteins discussed above, these proteins are also involved in controlling gene expression and regulating liquid–liquid phase separation-mediated compartmentalization, as well as an ever-growing list of other functions that could not be discussed here due to space limitations [178,179,180,181,182]. They have been implicated in a wide diversity of human diseases. Additionally, the discoveries of unexpected roles by DEAD/H-box proteins suggest that we are not yet close to having a complete understanding of their biology. Future studies on DEAD/H-box proteins will therefore be mechanistically informative and can lead to the development of novel therapies for a range of human diseases.

## Data Availability

Not applicable.

## Supplementary Materials

The following supporting information can be downloaded at: https://www.mdpi.com/article/10.3390/cells11101608/s1, Data S1: Phylogenetic analysis of human and mouse DEAD/H-box proteins; Data S2: List of human diseases associated with DEAD/H-box protein genes. References [183,184,185] are cited in the supplementary materials.
