# Supplementary material for: DEAD/H-Box Helicases in Immunity, Inflammation, Cell Differentiation, and Cell Death and Disease
Source: Cells. 2022 May 11;11(10):1608. doi: 10.3390/cells11101608 (PMC9139286; doi:10.3390/cells11101608)
Supplement: Supplementary file 1 [file cells-11-01608-s001.zip › cells-1687554-supplementary.pdf]

**Supplementary Data 1**: **Phylogenetic analysis of human and mouse DEAD/H-box proteins.** Human proteins have a suffix 'hum' and mouse proteins have a suffix 'mus' in their names. The evolutionary history was inferred using the Maximum Parsimony method. The bootstrap consensus tree inferred from 500 replicates is taken to represent the evolutionary history of the taxa analyzed [1]. Branches corresponding to partitions reproduced in less than 50% bootstrap replicates are collapsed. The MP tree was obtained using the Subtree-Pruning-Regrafting (SPR) algorithm with search level 1 in which the initial trees were obtained by the random addition of sequences (10 replicates) [2]. This analysis involved 480 amino acid sequences. The sequences were obtained from the InterPro database and parsed using a custom Python script. There were a total of 4019 positions in the final dataset. Evolutionary analyses were conducted in MEGA X [3].

**Supplementary Data 2**: **List of human diseases associated with DEAD/H-box protein genes.** The list was generated from the DisGeNET database web interface [4]. Individual tabs contain GDA evidence for individual genes.

**References**


1. Felsenstein, J. Confidence Limits on Phylogenies: An Approach Using the Bootstrap. *Evolution* **1985**, *39*, 783–791. https://doi.org/10.1111/j.1558-5646.1985.tb00420.x.
2. Nei, M.; Kumar, S. *Molecular Evolution and Phylogenetics*; Oxford University Press: New York, NY, USA, 2000.
3. Kumar, S.; Stecher, G.; Li, M.; Knyaz, C.; Tamura, K. MEGA X: Molecular Evolutionary Genetics Analysis across Computing Platforms. *Mol. Biol. Evol.* **2018**, *35*, 1547–1549. https://doi.org/10.1093/molbev/msy096.
4. Pinero, J.; Ramirez-Anguita, J.M.; Sauch-Pitarch, J.; Ronzano, F.; Centeno, E.; Sanz, F.; Furlong, L.I. The DisGeNET knowledge platform for disease genomics: 2019 update. *Nucleic Acids Res.* **2020**, *48*, D845–D855. https://doi.org/10.1093/nar/gkz1021.
