# Supplementary material for: DEAD/H-Box Helicases in Immunity, Inflammation, Cell Differentiation, and Cell Death and Disease
Source: Cells. 2022 May 11;11(10):1608. doi: 10.3390/cells11101608 (PMC9139286; doi:10.3390/cells11101608)

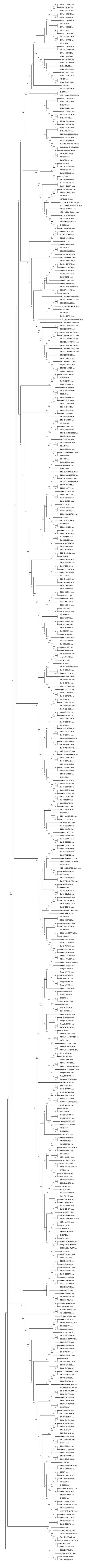

Supplement: Supplementary file 1 [file Supplementary Data 1.pdf › Supplementary Data 1.pdf]
